# Application of an Anomaly Detection Model to Screen for Ocular Diseases Using Color Retinal Fundus Images: Design and Evaluation Study

**DOI:** 10.2196/27822

**Published:** 2021-07-13

**Authors:** Yong Han, Weiming Li, Mengmeng Liu, Zhiyuan Wu, Feng Zhang, Xiangtong Liu, Lixin Tao, Xia Li, Xiuhua Guo

**Affiliations:** 1 Department of Epidemiology and Health Statistics, School of Public Health Capital Medical University Beijing China; 2 Beijing Municipal Key Laboratory of Clinical Epidemiology Capital Medical University Beijing China; 3 Department of Mathematics and Statistics La Trobe University Melbourne Australia

**Keywords:** anomaly detection, artificial intelligence, cataract, diabetic retinopathy, disease screening, eye, fundus image, glaucoma, macular degeneration, ocular disease, ophthalmology

## Abstract

**Background:**

The supervised deep learning approach provides state-of-the-art performance in a variety of fundus image classification tasks, but it is not applicable for screening tasks with numerous or unknown disease types. The unsupervised anomaly detection (AD) approach, which needs only normal samples to develop a model, may be a workable and cost-saving method of screening for ocular diseases.

**Objective:**

This study aimed to develop and evaluate an AD model for detecting ocular diseases on the basis of color fundus images.

**Methods:**

A generative adversarial network–based AD method for detecting possible ocular diseases was developed and evaluated using 90,499 retinal fundus images derived from 4 large-scale real-world data sets. Four other independent external test sets were used for external testing and further analysis of the model’s performance in detecting 6 common ocular diseases (diabetic retinopathy [DR], glaucoma, cataract, age-related macular degeneration, hypertensive retinopathy [HR], and myopia), DR of different severity levels, and 36 categories of abnormal fundus images. The area under the receiver operating characteristic curve (AUC), accuracy, sensitivity, and specificity of the model’s performance were calculated and presented.

**Results:**

Our model achieved an AUC of 0.896 with 82.69% sensitivity and 82.63% specificity in detecting abnormal fundus images in the internal test set, and it achieved an AUC of 0.900 with 83.25% sensitivity and 85.19% specificity in 1 external proprietary data set. In the detection of 6 common ocular diseases, the AUCs for DR, glaucoma, cataract, AMD, HR, and myopia were 0.891, 0.916, 0.912, 0.867, 0.895, and 0.961, respectively. Moreover, the AD model had an AUC of 0.868 for detecting any DR, 0.908 for detecting referable DR, and 0.926 for detecting vision-threatening DR.

**Conclusions:**

The AD approach achieved high sensitivity and specificity in detecting ocular diseases on the basis of fundus images, which implies that this model might be an efficient and economical tool for optimizing current clinical pathways for ophthalmologists. Future studies are required to evaluate the practical applicability of the AD approach in ocular disease screening.

## Introduction

Globally, approximately 2.2 billion people have vision impairment or blindness, according to the first World Report on Vision issued by the World Health Organization in 2019 [1]. In addition, the growth of the global population and the changes in its age structure are leading to accelerated growth of this number [2]. There is adequate evidence that retinal screening and referral for treatment can prevent avoidable blindness [3]. Advances in fundus imaging are expected to decrease preventable visual morbidity by enabling convenient and timely eye disease screening [4,5]. Color fundus camera imaging is an essential and easy-to-master technique for detecting a variety of eye diseases, such as diabetic retinopathy (DR) [6], age-related macular degeneration (AMD) [7], glaucoma [8], cataracts [9], and myopia [10,11]. However, in most countries, especially in low-income countries or regions with insufficient medical resources, there are not enough highly skilled ophthalmologists engaged in eye screening. Therefore, there is an urgent need to develop a convenient and low-cost auxiliary diagnostic system to screen for ocular diseases.

With the promotion of artificial intelligence (AI) in medicine over the past decade [12,13], AI has proven to be effective and feasible for automatic eye disease screening or diagnosis based on color retinal fundus images. Over the past few years, supervised deep learning approaches have provided state-of-the-art performance in eye disease classification tasks and have achieved excellent sensitivity and specificity in the detection of DR [14-16], AMD [17,18], cataracts [11], glaucoma [15,19], and pathological myopia [20]. However, these models may fail in real-life settings because they are trained on the basis of only one type of fundus disease, when a given fundus image used for inference is neither normal nor the specific ocular disease used in training; in such situations, the model is bound to make an incorrect prediction. A multiclass model that includes all types of ocular diseases may be an option to consider. However, such a model would require large data sets annotated by ophthalmologists, which is laborious and expensive. Furthermore, the gamut of all possible anomalies is not available in most cases owing to the rarity of certain diseases and disorders.

In disease screening, the proportion of normal samples is generally much higher than that of anomalies, which implies that the task is akin to anomaly detection (AD). In AD, a model is developed on the basis of only normal samples to capture the distribution of normality and is then evaluated on both unseen normal and abnormal samples to test their deviation from the distribution [21]. Numerous previous studies have focused on AD in understanding visual scenes [22-25], with a wide range of application domains [26-29]. However, AD has rarely been applied to medical images, where the distinctions between normality and anomaly may be subtler and more variable than those in natural images.

In the field of ophthalmology, Seeböck et al [30] developed the first AD system by training a 1-class support vector machine model unsupervised to identify anomalous regions in optical coherence tomography (OCT) images. Furthermore, a series of generative adversarial network (GAN)–based AD methods were proposed for OCT AD, which demonstrated excellent performance [31-33]. To date, only 1 study has adopted the isolation forest AD algorithm to detect ocular diseases on the basis of small-scale data sets of color retinal fundus images [34]. The area under the receiver operating characteristic curve (AUC) of the model for detecting premature retinopathy and DR were 0.770 and 0.745, respectively, which do yet meet clinical requirements. Hence, we decided to test the prospects of a deep learning–based AD model developed on a large-scale data set of color fundus images.

The primary aim of our study was to develop an AD model based on normal retinal fundus images for the detection of ocular diseases. Our secondary aim was to evaluate the performance of this approach in detecting 6 common ocular diseases, DR of different severity grades, and a variety of fundus abnormalities.

## Methods

### Methods Overview

**Figure 1:**
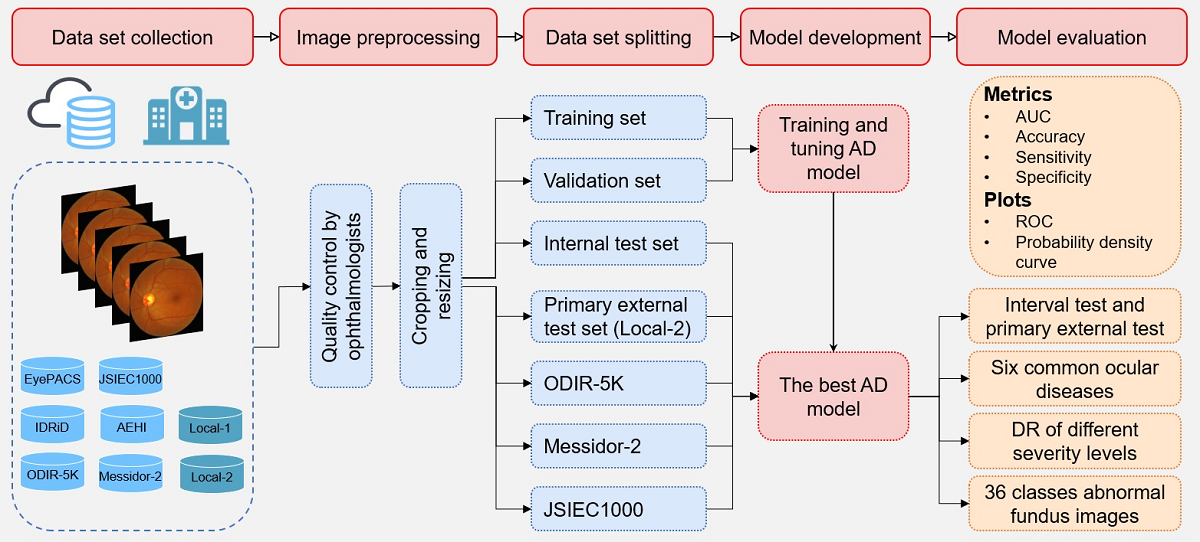
Workflow of this study. AD: anomaly detection, AUC: area under the receiver operating characteristic curve, DR: diabetic retinopathy, ODIR: Ocular Disease Intelligent Recognition, and ROC: receiver operating characteristic curve.

Figure 1 represents the workflow for the establishment and verification of the proposed AD method in this study. The first step was to compile fundus images from various sources into a large-scale data set, which contains public data sets available on websites and data sets of our own. The next step was to preprocess the fundus images, including manual removal of fundus images that are not qualified for diagnosis because of substandard quality, as well as cropping of images from different sources to a uniform size. In the third step, the AD model was developed on the training and validation sets. Specifically, the training set was applied to optimize the learnable parameters of the model, whereas the validation set was used to determine the best configuration of the hyperparameters (such as the learning rate, batch size, and momentum) through the random search strategy. The optimal hyperparameters refer to the combination of hyperparameters that can make the AD model achieve the highest AUC on the validation set. In the last step, the final model was first evaluated on internal and primary external test sets, and then we evaluated the model’s ability to detect 6 common ocular diseases, different severity levels of DR, and 36 types of fundus abnormal findings or diseases.

### Training, Validation, and Test Sets

Eight color fundus image data sets from various sources were collected for model development and evaluation in our study, which are described in Table 1. These fundus images were collected from numerous clinical or health care institutions with diverse models of color fundus cameras in 4 countries (United States, India, China, and France). All fundus images were centered near the macula, while the pupil dilation and field of vision were inconsistent.

We compiled 4 data sets (3 publicly available and 1 proprietary), which comprise 64,351 normal fundus images and 26,148 fundus images with lesions, as training, validation, and internal test sets. Since the model only needs normal fundus images for training, we randomly sampled 60% of normal fundus images as the training set, 20% of normal and 50% of abnormal fundus images as the validation set for hyperparameter tuning of the model during the training phase, and the remaining 20% of normal and 50% of abnormal fundus images as the test set for internal testing of the model’s performance.

The model was externally tested using 4 additional fundus image data sets, of which 1 is proprietary and the other 3 are publicly available (Table 1). Ocular Disease Intelligent Recognition (ODIR-5K) contains images of 6 common ocular diseases, namely DR (1334 diagnosable images), glaucoma (456 images), cataract (439 images), AMD (322 images), HR (251 images), and myopia (377 images). The Messidor-2 data set is a collection of DR examinations [35,36], which contains only abnormal images of DR, and each image is rated by a medical expert as 1 of 5 severity levels in accordance with the International Clinical Diabetic Retinopathy Disease Severity Scale [37]. The JSIEC1000 data set contains 36 categories of abnormal retinal fundus images, including some rare diseases such as retinitis pigmentosa, congenital disc abnormality, fundus neoplasm, and Vogt-Koyanagi-Harada disease.

We used these 4 data sets to further evaluate different aspects of model performance. The primary analysis was performed to evaluate the performance of the AD algorithm in detecting all abnormal fundus images in the Local-2 data set. Next, the following subsidiary analyses were performed: (1) the detection of 6 common ocular diseases was analyzed in the ODIR-5K data set, (2) the detection of different severities of DR was evaluated in the Messidor-2 data set, and (3) the detection of 36 abnormal findings or diseases was assessed on the basis of the JSIEC1000 data set.

**Table 1:** Summary of all data sets used to develop and evaluate the anomaly detection model.

Source data sets		Abbreviation	Race or ethnicity	Cohort	Camera models	Annotators	Number of diagnosable images	
							Normal	Abnormal
**Training, validation and internal test sets**								
	EyePACS program in California (United States)	EyePACS	Various ethnicities	Clinic-based	A variety of cameras	A panel of medical specialists	47,306	23,301
	Health Examination Center of Beijing Xiaotangshan Hospital (China)	Local-1	Chinese	Population-based	Topcon TRC-NW100	1 ophthalmologist	14,933	987
	Aravind Eye Hospital (India)	AEHI	Indian	Clinic-based	A variety of cameras	1 clinician	1764	1692
	Eye Clinic of Sushrusha Hospital (India)	IDRiD	Indian	Clinic-based	Unclear	Medical experts	348	168
**External test sets**								
	Beijing Physical Examination Center (China)	Local-2	Chinese	Population-based	Topcon TRC-NW400	1 ophthalmologist	17,027	1089
	More than 400 clinical hospitals in China (China)	ODIR-5K	Chinese	Clinic-based	A variety of cameras	Trained human readers	3492	3179
	Ophthalmology department of Brest University Hospital (France)	Messidor-2	French	Clinic-based	Topcon TRC NW6	1 medical expert	1017	731
	Joint Shantou International Eye Centre (China)	JSIEC1000	Chinese	Clinic-based	A variety of cameras	Medical experts	54	946

### Image Preprocessing

Our data sets were obtained from a wide range of real-world sources, and their characteristics reflect their origins; some images may be out of focus, of inaccurate exposure, or contain artefacts and noise that are not relevant to the diagnosis. For unsupervised AD methods, it is necessary to eliminate these low-quality fundus images to ensure that they are not recognized as abnormal images [38]. Two trained junior ophthalmologists (with 2~3 years of experience) were asked to identify and discard low-quality fundus images that were insufficient to make a reliable diagnosis independently. In case of discordance between these 2 screeners, arbitration was performed by a senior ophthalmologist (with 12 years of experience) to generate a final judgment. The number of diagnosable images in each data set is listed in Table 1.

Moreover, owing to the diverse fundus camera models and settings, the regions captured by the fundus photographs are also markedly heterogeneous, which may cause the model to learn features that are extraneous to disease diagnosis. Hence, we cropped all fundus images to save the same area and standardized the image sizes to a width and height of 800 pixels and 660 pixels, respectively. Multimedia Appendix 1 provides details regarding the pipeline for fundus image preprocessing. We determined the optimal input image resolution for loading into the model through a pilot study, which investigated image resolutions ranging from 32 × 32 pixels to 640 × 640 pixels. The results showed that an image resolution of 256 × 256 pixels achieved the maximum AUC (Multimedia Appendix 2). Hence, we reshaped the fundus images to 256 × 256 pixels when loading them into the AD model for training or inference.

### AD Algorithm

In this study, we adopted an AD method, known as Skip-GANomaly, proposed by Akçay et al [21]. This approach learns representations within both image and latent vector space jointly and achieves state-of-the-art performance both statistically and computationally.

**Figure 2:**
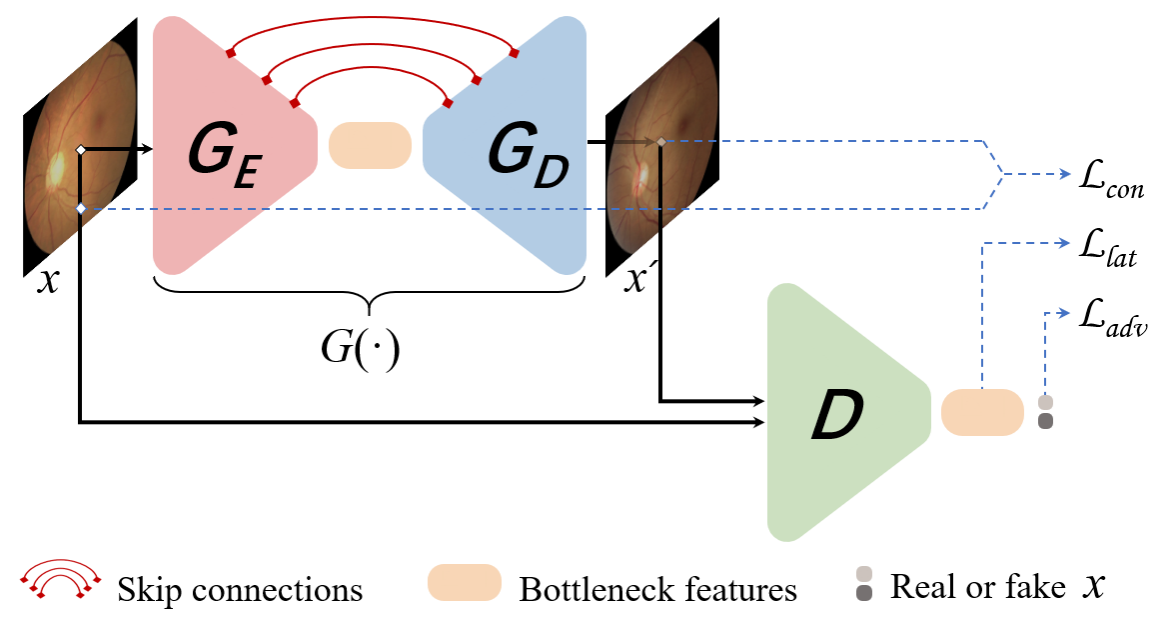
Overview of the training procedure of the Skip-GANomaly model.

As shown in Figure 2, this algorithm is a GAN network that includes a generator (G) and a discriminator (D) network. The generative network is similar to an automatic encoder, which comprises an encoder (G_E_) and a decoder (G_D_) network and skip connections between them. In the training phase, the model is trained only on normal samples, and the training objective is to capture the distribution of normal images within not only image space but also latent vector space. To achieve this goal, 3 loss equations are established: adversarial loss (*L*_adv_), contextual loss (*L_con_*), and latent loss (*L_lat_*). The total training objective becomes a weighted sum of the aforementioned losses*.* In the inference phase, an anomaly score composed of weighted contextual loss (*L_con_*) and latent loss (*L_lat_*) was used to detect the anomalies. The training objective would yield minimum anomaly scores for training samples (normal samples) but higher scores for abnormal images. Hence, a higher anomaly score for a given sample *x* indicates that *x* is likely abnormal with respect to the distribution of normal samples learned by the AD model from the training set during training [21]. The optimal threshold for the anomaly scores was determined using the Youden index [39]; that is, the critical threshold value that achieved the maximum Youden index was referred to as the optimal threshold.

We trained the AD model in an unsupervised manner on the training set (38,611 normal samples) and tuned the hyperparameters of the model on the validation set (containing 12,870 normal and 13,074 abnormal samples). The configuration of the optimal hyperparameters, the optimization objective of the model in the training phase, and the calculation method of anomaly score in the inference phase are detailed in Multimedia Appendix 3.

### Statistical Analysis

The metrics AUC, accuracy, sensitivity, and specificity were used to evaluate the performance of the AD algorithm. For the calculation, abnormal fundus images were regarded as positive samples, and normal fundus images were regarded as negative. The binomial exact 95% CI was calculated for the AUC, and the Wilson score was applied to calculate the Wilson 95% CIs for accuracy, sensitivity, and specificity. All statistical analyses were conducted using R (version 3.6.0, The R Foundation).

## Results

In total, the AD algorithm for detecting abnormal fundus images was developed using a training set of 38,611 normal fundus images and a validation set comprising 12,870 normal fundus images and 13,074 anomalies. With the optimal hyperparameters derived from model tuning, a final complete model was trained on all 51,481 images, including all normal fundus images in the training set and the validation set. Then, we evaluated the model’s performance in detecting abnormal fundus images in the internal and external test sets.

**Figure 3:**
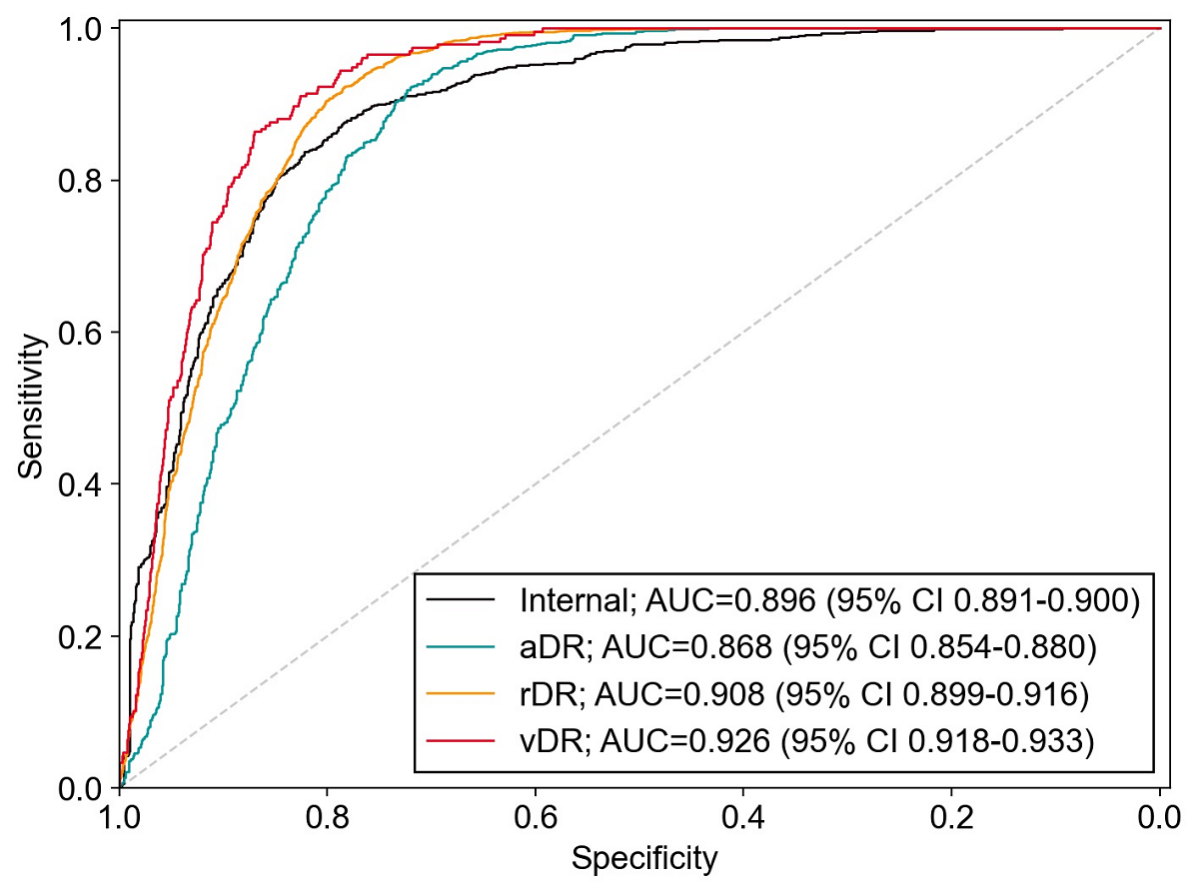
ROC and AUC of the anomaly detection model for detecting abnormalities in the internal test set, as well as detecting aDR, rDR, and vDR in the Messidor-2 data set. “internal” refers to internal test set. aDR diabetic retinopathy of any severity, AUC: area under the receiver operating characteristic curve, rDR: referable diabetic retinopathy, ROC: receiver operating characteristic, vDR vision-threatening diabetic retinopathy.

First, we investigated the model’s performance on the internal test set (containing 12,870 normal fundus images and 13,074 anomalies) and the external test set Local-2 (containing 17,027 normal fundus images and 1089 anomalies). The algorithm achieved an AUC of 0.896 on the internal test set (Table 2 and Figure 3). The maximum Youden index was 0.6536 (sensitivity=82.69% and specificity=82.63%), which corresponded to the optimal threshold of 2.874×10^-3^. The model’s performance on external test set Local-2 was equal to its performance on the internal test set (Table 2).

**Table 2:** The performance of the anomaly detection model in detecting abnormal fundus images in the internal and external test sets.

Test data sets	Area under the receiver operating characteristic curve (95% CI)^a^	Proportion (%) (95% CI)^b^		
		Accuracy	Sensitivity	Specificity
Internal test set	0.896 (0.891- 0.900)	82.67 (82.10-83.22)	82.69 (81.94-83.42)	82.63 (81.74-83.49)
Local-2	0.900 (0.893-0.906)	83.35 (82.63-84.04)	83.25 (82.52-83.97)	85.19 (81.90-88.07)

^a^The binomial exact 95% CI was calculated for each are under the receiver operating characteristic curve.

^b^The Wilson score was applied to calculate the Wilson 95% CI for accuracy, sensitivity, and specificity.

**Figure 4:**
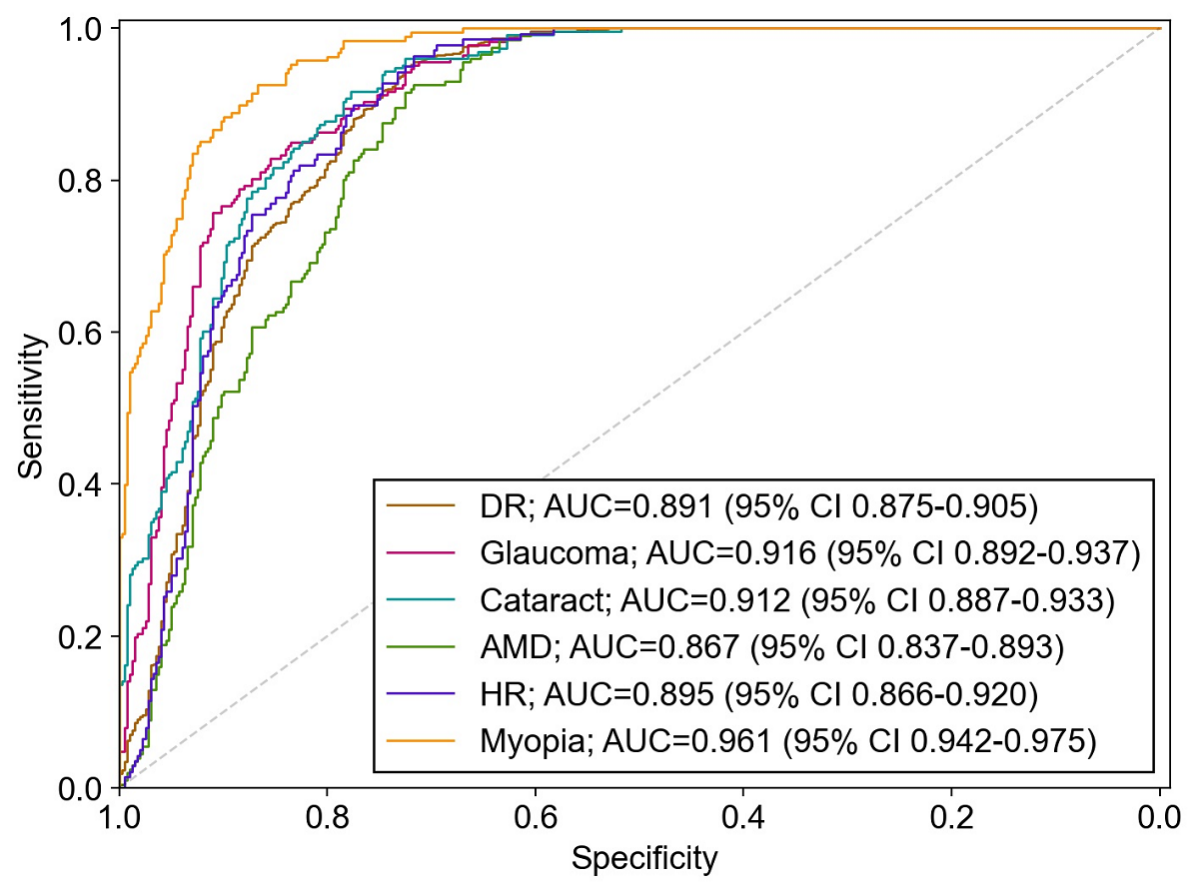
ROC and AUC of the anomaly detection model for detecting 6 ocular diseases. AMD: age-related macular degeneration, AUC: area under the receiver operating characteristic curve, DR: diabetic retinopathy, HR: hypertensive retinopathy, and ROC: receiver operating characteristic curve.

**Figure 5:**
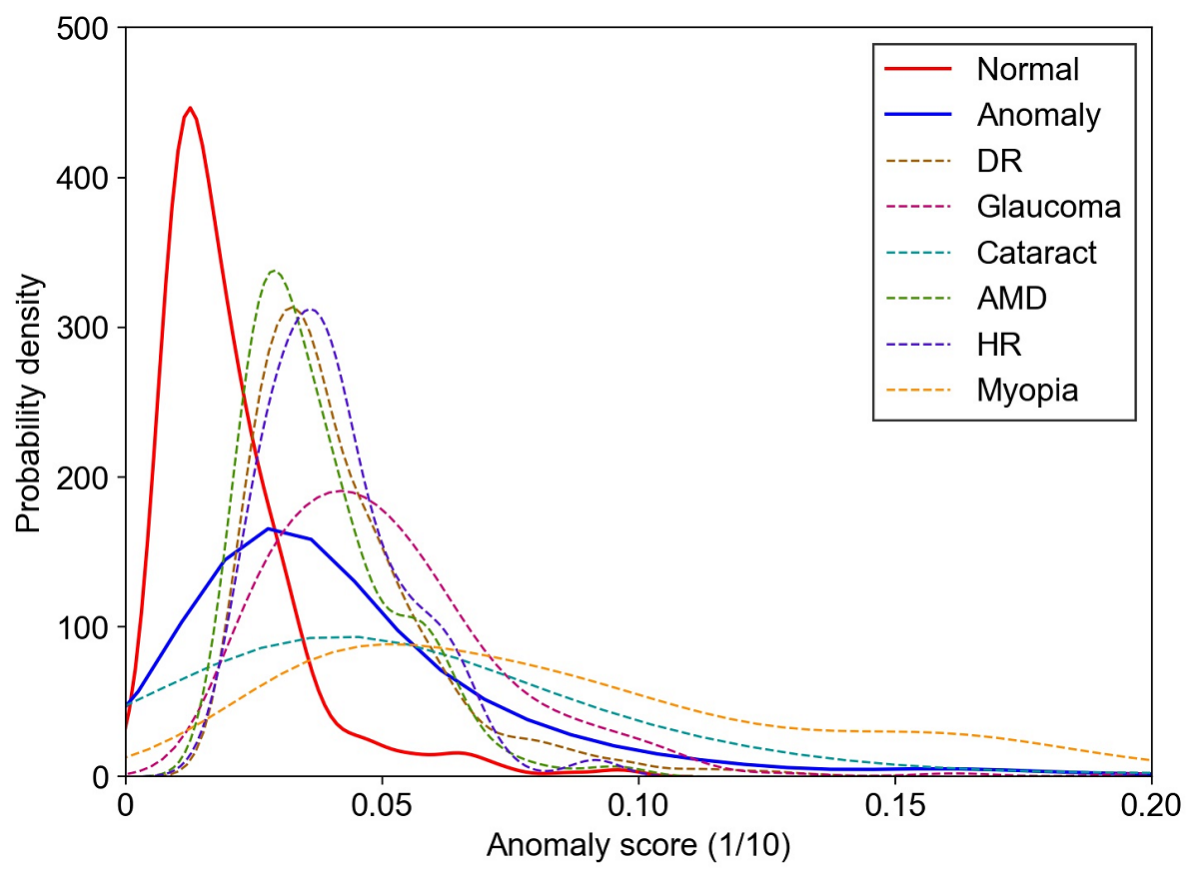
The probability density curves of the anomaly scores for the Ocular Disease Intelligent Recognition-5K test set. The solid red curve indicates the anomaly score distribution of normal fundus images, and the solid blue curve indicates the anomaly score distribution of all abnormal fundus images. The dotted line represents the anomaly score distribution of 6 types of abnormal fundus images. AMD: age-related macular degeneration, and DR: diabetic retinopathy, HR: hypertensive retinopathy.

Next, to explore the model’s capacity to detect different ocular diseases, we evaluated the model’s effectiveness in detecting 6 common types of ocular diseases by using the ODIR-5K data set (Table 3 and Figure 4). The top 3 AUCs were 0.961, 0.916, and 0.912, which corresponded to myopia, glaucoma, and cataract, respectively. AMD had the lowest AUC of 0.867 among all 6 categories. The model had greater than 80% accuracy, sensitivity, and specificity for the detection of all evaluated ocular diseases, except for AMD. Furthermore, we calculated the anomaly score of each fundus image and plotted the distribution of the scores by using probability density curves (Figure 5).

**Table 3:** The Ocular Disease Intelligent Recognition-5K test set: area under the receiver operating characteristic curve, accuracy, sensitivity, and specificity of the anomaly detection model in detecting 6 ocular diseases.

Ocular diseases	Area under the receiver operating characteristic curve (95% CI)^a^	Proportion (%) (95% CI)^b^		
		Accuracy	Sensitivity	Specificity
All anomalies	0.896 (0.885-0.906)	80.72 (79.39-81.99)	80.69 (79.27-82.03)	81.00 (76.87-84.54)
Diabetic retinopathy	0.891 (0.875-0.905)	80.39 (78.42-82.23)	80.36 (78.09-82.45)	80.50 (76.33-84.08)
Glaucoma	0.916 (0.892-0.937)	83.89 (80.81-86.56)	83.70 (78.34-87.94)	84.00 (80.09-87.27)
Cataract	0.912 (0.887-0.933)	83.44 (80.33-86.14)	83.33 (77.95-87.61)	83.50 (79.55-86.82)
Age-related macular degeneration	0.867 (0.837-0.893)	78.37 (74.90-81.48)	77.61 (71.36-82.83)	78.75 (74.48-82.48)
Hypertensive retinopathy	0.895 (0.866-0.920)	82.37 (78.93-85.36)	81.29 (74.00-86.90)	82.75 (78.74-86.14)
Myopia	0.961 (0.942-0.975)	88.78 (85.97-91.08)	88.83 (83.53-92.58)	88.75 (85.28-91.49)

^a^The binomial exact 95% CI was calculated for each are under the receiver operating characteristic curve.

^b^The Wilson score was applied to calculate the Wilson 95% CI for accuracy, sensitivity, and specificity.

Then, we assessed the performance of the model in detecting fundus images of referable DR and vision-threatening DR by using the Messidor-2 data set. Referable DR was defined as moderate DR or worse (severe DR and proliferative DR), and vision-threatening DR was defined as severe or proliferative DR. The AUC of the AD model was 0.908 for referable DR and 0.926 for vision-threatening DR, both of which were higher than the AUC of 0.868 for any DR (Table 4 and Figure 3).

Finally, we evaluated the model’s performance in detecting 36 abnormal findings or diseases on retinal fundus images by using the JSIEC1000 data set. In detecting any abnormal findings or diseases, the model’s AUC was 0.895 with an accuracy of 82.35%, sensitivity of 82.69%, and specificity of 82.99%. For each of the 36 abnormal findings or diseases, the AUC values ranged from 0.630 to 0.987, in which 23 of 36 categories had AUCs greater than 0.9, 9 categories had AUCs ranging from 0.8 to 0.9, and 4 categories had AUCs less than 0.8 (Multimedia Appendix 4).

**Table 4:** The Messidor-2 test set: area under the receiver operating characteristic curve, accuracy, sensitivity, and specificity of the anomaly detection model in detecting diabetic retinopathy of different severities.

Severity of diabetic retinopathy	Area under the receiver operating characteristic curve (95% CI)^a^	Proportion (%) (95% CI)^b^		
		Accuracy	Sensitivity	Specificity
Any diabetic retinopathy	0.868 (0.854-0.880)	79.45 (78.36-80.52)	79.47 (78.12-80.77)	79.41 (77.49-81.24)
Referable diabetic retinopathy	0.908 (0.899-0.916)	83.38 (81.49-84.84)	83.36 (81.77-84.84)	83.39 (81.87-84.81)
Vision-threatening diabetic retinopathy	0.926 (0.918-0.933)	86.40 (81.45-88.19)	86.38 (81.41-90.19)	86.43 (85.02-87.73)

^a^The binomial exact 95% CI was calculated for each are under the receiver operating characteristic curve.

^b^The Wilson score was applied to calculate the Wilson 95% CI for accuracy, sensitivity, and specificity.

## Discussion

### Principal Findings

In this study, a GAN-based AD approach was developed to detect ocular diseases or abnormalities by using only normal fundus images. Four data sets containing over 53,236 color fundus images from various geographic and ethnic groups were applied for model training, validation, and internal testing, along with 4 data sets for external testing. Our results show that our approach achieved an AUC of 0.896, sensitivity of 80.69%, and specificity of 81.00% in detecting abnormal fundus images in the internal test set, and the developed model showed stable and consistent performance in the primary external test set, Local-2. This study further analyzed the model’s ability to detect DR, glaucoma, cataracts, AMD, HR, and myopia and compared the performance of the model in detecting different severities of DR. To our knowledge, this is the first study using large-scale data sets of color fundus images to detect various ocular diseases by using an AD approach. The contribution of the AD model we established is to automatically detect abnormal fundus images so that the ophthalmologist can focus on the diagnosis based on abnormal fundus images and avoid spending extensive time and effort on masses of normal fundus images.

### Comparison With Existing Studies

Previous studies have applied AD to screen eye disorders; however, most of those studies were based on OCT images. Retinal fundus photography is simpler to operate and more cost-effective than OCT, which renders it suitable for early screening of ocular disease [40]. Thus far, only Ouardini et al [34] have used AD with regard to color retinal fundus images; however, the 2 data sets they used were small, and only 1 type of fundus abnormality (retinopathy of prematurity or DR) was included in each data set. In our study, we developed an AD model based on 4 large-scale data sets derived from clinical or population screening and conducted external validation on 4 independent data sets, which ensured the robustness and generalizability of the model. Hence, this study substantially complements the findings of previous studies.

In recent years, there has been a surge in supervised deep learning studies for classifying fundus images, and many studies have achieved excellent performance. For example, Ting et al [41] trained a deep learning system to detect specific ocular diseases through binary classification tasks, achieving 90.5% sensitivity and 91.6% specificity for the detection of referable DR, 96.4% sensitivity and 87.2% specificity for possible glaucoma, and 93.2% sensitivity and 88.7% specificity for AMD, which is markedly higher than those of our method. The main reason may be that we used unsupervised learning; the model only learns the distribution pattern of normal images in model training, rather than the distributions of normal and abnormal images simultaneously as in supervised learning [42].

However, the unsupervised AD approach applied in our study has several specific advantages. First, model fitting does not require any labeled abnormal fundus images for training, which greatly reduces the cost of image annotation. Second, the AD model from our study can theoretically detect all classes of abnormal fundus images, including those of rare ocular diseases, while supervised learning is limited to detecting the types of abnormal images used in model training. Third, owing to the existence of various types of abnormal fundus images in clinical practice, the applicability of binary classification models is very low. For instance, the premise for a binary classification model to be applicable for distinguishing between normal and DR images is that the fundus image to be discriminated by the model must be either a normal fundus image or a DR fundus image. Any other type of image will inevitably lead to incorrect classification results. However, AD models do not present such issues.

### Limitations

This study has some limitations of note. First, the model we established in this study has lower precision than that achievable by a supervised learning model. Nevertheless, the detection performance of the AD algorithm remained clinically acceptable and highly reproducible both in the internal test set and in external data sets. For DR screening, international guidelines recommended a minimum sensitivity of 60% (Australia) to 80% (United Kingdom) [43,44]. In clinical applications, to ensure that the model has a minimal false-negative rate, the sensitivity of the model can be increased by lowering the threshold of anomaly scores. Second, this model can only screen out abnormal fundus images and cannot directly provide specific diagnoses, which still require an ophthalmologist for completion. As such, the model is not capable of making a fully automated diagnosis, and its primary function is to allow ophthalmologists to focus more on the fundus images of possible lesions. The AD model is well suited for fundus screening in the general population, which predominately includes normal fundus images. Owing to the wide variety of existing ocular diseases, the use of fundus images alone is not a sufficient basis for accurate diagnosis of ocular diseases, and the model also requires information regarding the patient’s medical history and clinical findings. Furthermore, the AD approach can detect anomalies only at the image level, not at the pixel level, and cannot show the specific locations of anomalies (eg, hard exudate, retinal vein occlusion, and macular hole) with heatmaps. The inherent black-box nature of deep learning may affect its acceptance for clinical use by ophthalmologists [45].

### Conclusion

In conclusion, we developed and evaluated a cost-effective and time-efficient AD model to screen for ocular diseases or abnormalities, which showed high sensitivity and specificity. Further studies are required to determine the feasibility of applying this algorithm for clinical diagnosis or screening and to determine whether the use of this algorithm could lead to improved care and outcomes, compared to the current diagnostic workflow.

## Appendix

Multimedia Appendix 1 Pipeline of image pre-processing.

Multimedia Appendix 2 Comparison of area under the receiver operating characteristic curve (AUC) with varying input image resolution for AD model.

Multimedia Appendix 3 Modeling and inference scheme of the Skip-GANomaly algorithm.

Multimedia Appendix 4 The anomaly detection performance of Skip-GANomaly model for 36 categories abnormal fundus image using JISEC1000 dataset.

## Abbreviations

AD: anomaly detection
AI: artificial intelligence
AMD: age-related macular degeneration
AUC: area under the receiver operating characteristic curve
DR: diabetic retinopathy
GAN: generative adversarial network
HR: hypertensive retinopathy
OCT: optical coherence tomography
ODIR: Ocular Disease Intelligent Recognition
