# Supplementary material for: Application of an Anomaly Detection Model to Screen for Ocular Diseases Using Color Retinal Fundus Images: Design and Evaluation Study
Source: J Med Internet Res. 2021 Jul 13;23(7):e27822. doi: 10.2196/27822 (PMC8317033; doi:10.2196/27822)
Supplement: Multimedia Appendix 1 [file jmir_v23i7e27822_app1.docx]

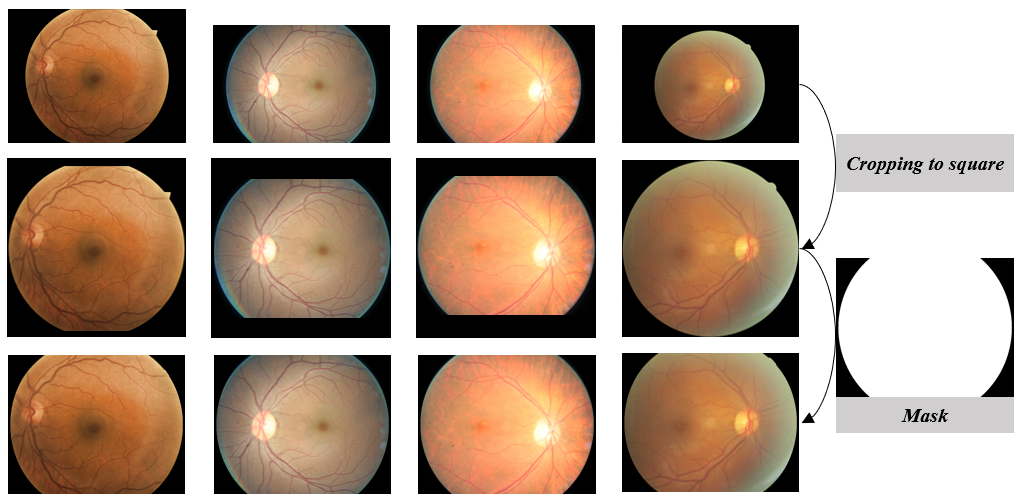
**FIGURE.** Pipeline of image pre-processing. Firstly, the Hough circle detection algorithm was used to detect the circular edge in the fundus image, and the square region containing the central circle was cut out according to the edge. Then applied a mask to obtain the common region of interest of all fundus images.
