# Supplementary material for: Application of an Anomaly Detection Model to Screen for Ocular Diseases Using Color Retinal Fundus Images: Design and Evaluation Study
Source: J Med Internet Res. 2021 Jul 13;23(7):e27822. doi: 10.2196/27822 (PMC8317033; doi:10.2196/27822)
Supplement: Multimedia Appendix 2 [file jmir_v23i7e27822_app2.docx]

**
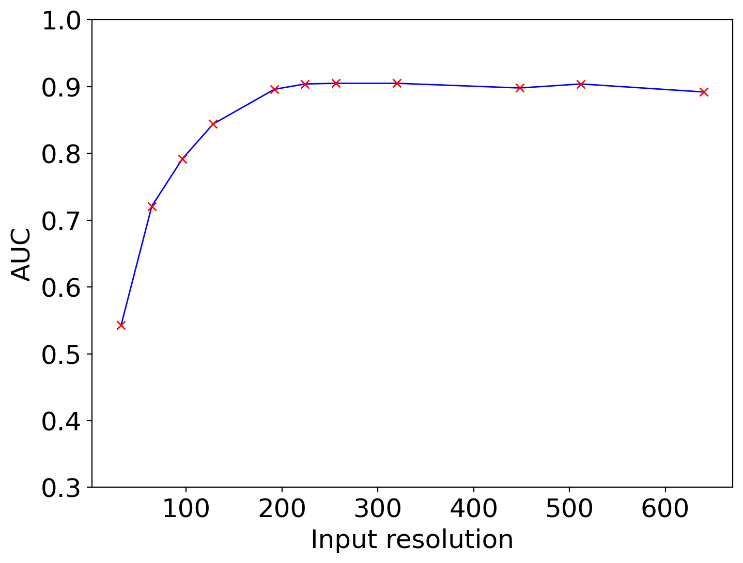
**

FIGURE. Comparison of area under the receiver operating characteristic curve (AUC) with varying input image resolution for AD model. The plot showed that AUCs show improved performance with increased image resolution and a plateau effect on performance improvement for resolutions higher than 256 × 256 pixels. Resolutions and corresponding AUC values shown are as follows: 32 × 32 (AUC: 0.543), 64 × 64 (AUC: 0.721), 96 × 96 (AUC: 0.792), 128 × 128 (AUC: 0.844), 192 × 192 (AUC: 0.896), 224 × 224 (AUC: 0.904), 256 × 256 (AUC: 0.905), 320 × 320 (AUC: 0.904), 448 × 448 (AUC: 0.898), 512 × 512 (AUC: 0.904), and 640 × 640 (AUC: 0.892) pixels.
