# Supplementary material for: Application of an Anomaly Detection Model to Screen for Ocular Diseases Using Color Retinal Fundus Images: Design and Evaluation Study
Source: J Med Internet Res. 2021 Jul 13;23(7):e27822. doi: 10.2196/27822 (PMC8317033; doi:10.2196/27822)
Supplement: Multimedia Appendix 3 [file jmir_v23i7e27822_app3.docx]

**Modeling and inference scheme of the Skip-GANomaly algorithm^1^**

***A. Training Objective***The idea of Skip-GANomaly algorithm is to train the model only on normal samples, and test on both normal and abnormal ones. We expect the model to be able to correctly reconstruct the normal samples either in image or latent vector space. The hypothesis is that the network is conversely expected to fail to reconstruct the abnormal samples as it is never trained on such abnormal examples. Hence, for abnormal samples, one would expect a higher loss for the reconstruction of the output image representation $\hat{x}$ or the latent representation $\hat{z}$. Skip-GANomaly algorithm proposes to combine three loss values (*Adversarial, Contextual, Latent*), each of which has its own contribution to make within the overall training objective.

*1) Adversarial Loss:* In order to maximize the reconstruction capability for the normal images *x* during training, Skip-GANomaly algorithm utilizes an adversarial loss. This loss, shown in Equation 1, ensures that the network *G* reconstructs a normal image *x* to $\hat{x}$ as realistically as possible, while the discriminator network *D* classifies the real and the (fake) generated samples. The task here is to minimize this objective for *G*, and maximize for *D* to achieve min *G* max *D L_adv_*, where *L_adv_* is denoted as

$\mathcal{L}_{adv}=\underset{x\sim p_{x}}{\mathbb{E}}[\log D(x)]+\underset{x\sim p_{x}}{\mathbb{E}}[\log(1-D(\hat{x})]$ (1)

*2)* *Contextual Loss:* The adversarial loss imposes the model to generate realistic samples, but does not guarantee to learn contextual information regarding the input. To explicitly learn this contextual information to sufficiently capture the input data distribution for the normal samples, Skip-GANomaly algorithm applies *L*_1_ normalization to the input *x* and the reconstructed output $\hat{x}$. This normalization ensures that the model is capable of generating contextually similar images to normal samples. The contextual loss of the training objective is shown below:

$\mathcal{L}_{con}=\underset{x\sim p_{x}}{\mathbb{E}}|x-\hat{x}|_{1}$ (2)

*3) Latent Loss:* With the adversarial and contextual losses defined above, the model is able to generate realistic and contextually similar images. In addition to these objectives, Skip-GANomaly algorithm aims to reconstruct latent representations for the input *x* and the generated normal samples $\hat{x}$ as similar as possible. This is to ensure that the network is capable of producing contextually sound latent representations for common examples. As depicted in **Figure 1 of main text**, this model uses the final convolutional layer of the discriminator *D*, and extract the features of *x* and $\hat{x}$ to reconstruct their latent representations such that *z* = *f*(*x*) and $\hat{z}$ = *f*($\hat{x}$). The latent representation loss therefore becomes:

$\mathcal{L}_{lat}=\underset{x\sim p_{x}}{\mathbb{E}}|f(x)-f( \hat{x} )|_{2}$ (3)

Finally, total training objective becomes a weighted sum of the losses above.

$\mathcal{L=}\lambda_{adv}\mathcal{L}_{adv}+\lambda_{con}\mathcal{L}_{con}+\lambda_{lat}\mathcal{L}_{lat}$ (4)

where $\lambda_{adv}$, $\lambda_{con}$ and $\lambda_{lat}$ are the weighting parameters adjusting the dominance of the individual losses to the overall objective function.

***B. Training***

The training objective $\mathcal{L}$ from Equation 4 is optimized via Adam optimizer with an initial learning rate *lr* = 1*e^-^*^3^ with a lambda decay, and momentums *β*_1_ = 0.5, *β*_2_ = 0.999. The batch size is 4, number of extractor layers of network *G* and *D* is 8, and size of the latent z vector is 100. The weighting parameters of $\mathcal{L}$ is chosen as $\lambda_{adv}$ = 1, $\lambda_{con}$ = 15 and $\lambda_{lat}$ = 1, empirically shown to yield the optimal performance. The model is initially set to be trained for 45 epochs; however, in most cases it learns sufficient information within less training cycles. Therefore, we save the parameters of the network when the performance of the model starts to decrease since this reduce is a strong indication of over-fitting. The model is implemented using PyTorch (v0.4.0, Python 3.6.9, CUDA 9.2 and CUDNN 7.1). Experiments are performed using an NVIDIA Quadro P1000 GPU.

***C. Inference***Anomaly score is employed to find the anomalies during the testing and subsequent deployment. For a given test image $\dot{x}$, its anomaly score becomes:

$\mathcal{A(}\dot{x})=\lambda R(\dot{x})+(1-\lambda)L(\dot{x})$ (5)

where $R(\dot{x})$ is the reconstruction score measuring the contextual similarity between the input and the generated images based on Equation 2. $L(\dot{x})$ denotes the latent representation score measuring the difference between the input and generated images based on Equation 3. *λ* is the weighting parameter controlling the relative importance of the score functions, which is 0.9 in this study.

**Reference**

1. Akçay S, Atapour-Abarghouei A, Breckon TP. Skip-GANomaly: Skip Connected and Adversarially Trained Encoder-Decoder Anomaly Detection. 2019:1-8.
