# Supplementary material for: Application of an Anomaly Detection Model to Screen for Ocular Diseases Using Color Retinal Fundus Images: Design and Evaluation Study
Source: J Med Internet Res. 2021 Jul 13;23(7):e27822. doi: 10.2196/27822 (PMC8317033; doi:10.2196/27822)
Supplement: Multimedia Appendix 4 [file jmir_v23i7e27822_app4.docx]

**TABLE.** The anomaly detection performance of Skip-GANomaly model for 36 categories abnormal fundus image using JISEC1000 dataset.

| Disease | AUC | Accuracy, % | Sensitivity, % | Specificity, % |
| --- | --- | --- | --- | --- |
| Large optic cup | 0.963 | 93.16 | 93.65 | 92.59 |
| BRVO | 0.911 | 82.65 | 81.82 | 83.33 |
| CRVO | 0.939 | 88.16 | 86.36 | 88.89 |
| RAO | 0.964 | 91.43 | 87.50 | 92.59 |
| RRD | 0.966 | 92.79 | 92.98 | 92.59 |
| CSCR | 0.981 | 97.06 | 92.86 | 98.15 |
| Maculopathy | 0.857 | 77.34 | 77.03 | 77.78 |
| ERM | 0.976 | 95.00 | 96.15 | 94.44 |
| MH | 0.735 | 67.53 | 65.22 | 68.52 |
| Pathological myopia | 0.957 | 91.67 | 90.74 | 92.59 |
| Possible glaucoma | 0.987 | 97.01 | 92.31 | 98.15 |
| Disc swelling and elevation | 0.838 | 80.60 | 69.23 | 83.33 |
| Dragged Disc | 0.981 | 96.88 | 90.00 | 98.15 |
| Congenital disc abnormality | 0.896 | 68.75 | 80.00 | 66.67 |
| Retinitis pigmentosa | 0.955 | 92.11 | 90.91 | 92.59 |
| Peripheral retinal degeneration | 0.979 | 95.59 | 92.86 | 96.30 |
| Myelinated nerve fiber | 0.918 | 81.54 | 72.73 | 83.33 |
| Asteroid hyalosis | 0.771 | 69.12 | 64.29 | 70.37 |
| Fundus neoplasm | 0.981 | 96.77 | 87.50 | 98.15 |
| Hard exudates | 0.866 | 85.07 | 76.92 | 87.04 |
| Yellow-white spots | 0.898 | 78.57 | 76.67 | 79.63 |
| Cotton-wool spots | 0.887 | 86.15 | 72.73 | 88.89 |
| Chorioretinal atrophy-coloboma | 0.933 | 94.20 | 86.67 | 96.30 |
| Preretinal hemorrhage | 0.939 | 82.81 | 80.00 | 83.33 |
| Fibrosis | 0.854 | 89.06 | 70.00 | 92.59 |
| Laser Spots | 0.913 | 82.43 | 80.00 | 83.33 |
| Silicon oil in eye | 0.810 | 73.97 | 68.42 | 75.93 |
| Blur fundus without PDR | 0.777 | 70.00 | 69.81 | 70.37 |
| Blur fundus with suspected PDR | 0.975 | 94.29 | 94.12 | 94.44 |
| Bietti crystalline dystrophy | 0.981 | 96.77 | 87.50 | 98.15 |
| Optic atrophy | 0.943 | 87.88 | 83.33 | 88.89 |
| VKH | 0.630 | 60.29 | 50.00 | 62.96 |
| Tessellated fundus | 0.866 | 75.47 | 76.92 | 74.07 |
| Background DR | 0.907 | 81.25 | 78.57 | 83.33 |
| Pre-proliferative DR | 0.925 | 86.79 | 84.62 | 88.89 |
| Proliferative DR | 0.944 | 87.72 | 86.67 | 88.89 |

Abbreviations: BRVO, branch retinal vein occlusion; CRVO, central retinal vein occlusion; RAO, retinal artery occlusion; RRD, rhegmatogenous retinal detachment; CSCR, central serous chorioretinopathy; ERM, epiretinal gembrane; MH, macular hole; VKH, Vogt-Koyanagi-Harada disease; DR, diabetic retinopathy.
